# Consumer perspective from people with a diagnosis of Borderline Personality Disorder (BPD) on BPD management—How are the Australian NHMRC BPD guidelines faring in practice?

**DOI:** 10.1111/jpm.12714

**Published:** 2020-11-30

**Authors:** Jessica Margot Proctor, Sharon Lawn, Janne McMahon

**Affiliations:** ^1^ Behavioural Health, College of Medicine and Public Health Flinders University Adelaide SA Australia; ^2^ Lived Experience Australia Marden SA Australia

**Keywords:** Borderline Personality Disorder, health services delivery, patient experience, quality of care, stigma

## Abstract

**What is known on the subject?:**

- Internationally, stigma towards people with mental illness has reduced due to greater understanding, education and advocacy in the community, and more focus on recovery‐oriented care within practice guidelines.
- However, many people with a diagnosis of BPD continue to experience stigma and difficulty accessing health services. Contributing factors include lack of understanding of BPD and effective management by health professionals, stigma from the general population and within healthcare services, and financial and geographical barriers.
- Mental health nurses comprise a large part of the healthcare workforce responsible for the day‐to‐day care of people diagnosed with BPD.

**What the paper adds to existing knowledge:**

- This paper investigates how Australian consumer perspectives on BPD management have changed over time. Comments from a large survey, delivered to consumers in 2011 (*N* = 153) and 2017 (*N* = 424), were analysed for common themes.
- Themes were broadly related to NHMRC BPD Guidelines sections released in 2013. These data sets therefore present an opportunity to evaluate changes in consumer perspectives pre‐ and post‐Guideline release. Although no direct causal relationship can be drawn, analysing these changes can potentially assist with understanding the impact of the Guidelines in practice. No such analysis of the Australian Guidelines has been conducted to date in the existing literature.

**What are the implications for practice?:**

- Many people diagnosed with BPD continue to experience stigma, barriers to treatment and difficulty accessing appropriate services.
- Widespread practical implementation of the Guidelines was not apparent; however, improved general awareness and understanding of BPD from consumers and health professionals were evident. Improved education and practice across each and all aspects of the Guidelines are indicated.
- The Guidelines need review to ensure they are in‐line with current evidence‐based practice, as well as effective health professional education, support and funding to embed the revised Guidelines into practice.

**Abstract:**

**Introduction**

Internationally, many individuals diagnosed with BPD continue to experience stigma within health care and are more likely to be viewed as manipulative and evoke negative responses from clinicians, compared with other mental health consumers.

**Aim/Question**

To understand Australian consumer perspectives regarding BPD management, and how these have changed between 2011 and 2017. To comment on how NHMRC BPD Guidelines, released 2013, are faring in practice.

**Method**

Individuals who identified a BPD diagnosis completed a 75‐question survey, delivered online Australia‐wide, in 2011 (*N* = 153) and 2017 (*N* = 424), providing comparative data sets to evaluate changes in consumer perspective on BPD management.

**Results**

Many people diagnosed with BPD experience difficulties when seeking help, stigma within health services and barriers to treatment. Improved general awareness, communication and understanding of BPD from consumers and health professionals were evident.

**Discussion**

Consumers demonstrated increased BPD‐literacy and help‐seeking behaviours in 2017, providing opportunity for health professionals to build stronger therapeutic relationships. Widespread practical implementation of the Guidelines does not appear to have been achieved. More health professional education, updated Guidelines, funding and strategies to embed these Guidelines into practice are needed.

**Implications for Practice**

Mental health nurses regularly provide care to people diagnosed with BPD; with practical education and support, they and other health professionals can improve their confidence in practice and provide better quality care to consumers.

## 1 INTRODUCTION

Borderline personality disorder (BPD) is now accepted as a valid psychiatric diagnosis with specific and effective psychotherapeutic treatments available, but this has not always been the case (Gunderson, 2009). Internationally, individuals diagnosed with BPD still experience increased levels of stigma, are more likely to be viewed as manipulative and evoke negative responses from health professionals more frequently, compared with individuals with other mental health diagnoses (Liebman & Burnette, 2013; Ring & Lawn, 2019; Sansone & Sansone, 2013). Prior to 1970, the term “borderline” was used colloquially among psychiatrists, internationally, to refer to patients who had untreatable neuroses and psychoses that did not fit other diagnoses (Gunderson, 2009). BPD was not included as an official diagnosis in the Diagnostic and Statistical Manual for Mental Disorders until 1980 in DSM‐III (Gunderson, 2009). Since then, increasing research has been conducted to define the characteristics of BPD and address the needs of individuals with this disorder; however, in Australia, people with this diagnosis continue to have among the highest levels of unmet needs in terms of access to suitable, evidence‐based mental health services (NHMRC, 2013; NHMRC, 2013). Previous research has discussed the negative effects of stigma on these individuals; as people with a BPD diagnosis are often highly sensitized to rejection and abandonment, if they perceive this from treating health professionals, they can respond with self‐harm and withdrawal from treatment (Aviram et al., 2006; Kling, 2014).

This complex set of circumstances has led to uncertainty and controversy amongst mental health professionals in disclosing a diagnosis of BPD to an individual, especially adolescents, due to concern for potential stigmatizing effects (Ring & Lawn, 2019; Wlodarczyk et al., 2018). However, recent research has shown that disclosing the diagnosis to the individual assists them in understanding their experiences and in receiving effective treatment (Courtney & Makinen, 2016; Kaess et al., 2014). Transparency regarding an individual's diagnosis of BPD can be positive for several reasons, including respecting their autonomy, avoiding misinformation via the Internet, ensuring they receive suitable treatment and reassuring them that their distress is due to a known illness, with effective treatments available (Lequesne & Hersh, 2004). Additionally, prompt and clear communication to the consumer regarding their diagnosis can avoid later disengagement with treatment, as demonstrated in a study by Sulzer et al. (2016), “patients who later discovered that their diagnosis had been withheld consistently left treatment.” This research examined 32 patients and 32 clinicians, who were interviewed regarding communication of BPD diagnoses. The majority of clinicians chose not to disclose a BPD diagnosis to a patient, even when they felt it was most appropriate, usually motivated by a desire to avoid the stigma associated with a BPD diagnosis. This is in direct contrast with most patients, who wanted to be told their diagnosis. Of note is that most patients in the sample wanted to specifically discuss the stigma they may face with their clinician. Patients in the sample overwhelmingly reported relief at being given their diagnosis, to the surprise of the clinicians in the sample. This research not only highlights the essential nature of open communication in a therapeutic relationship, but also highlights the extent to which stigma towards people with a diagnosis of BPD is manufactured from within mental health services. A study by Zanarini and Frankenburg (2008) also demonstrated that individuals who are educated about BPD with up‐to‐date information immediately after diagnosis experience significantly lower short‐term impulsivity and interpersonal relationship distress. The recent SANE report (Carrotte et al., 2019) further highlights the problems with stigma from within health services, and the negative effects of unclear or insensitive communication from health professionals in the Australian setting.

Placing trust in health professionals can be especially challenging for people with a diagnosis of BPD, for a multitude of reasons. Looking at Ward's (2017) paper on trust in public health, two different kinds of trust are outlined. He takes Sabel's (1993) definition of interpersonal trust; “the mutual confidence that no party will exploit another's vulnerability,” and extends this to address the power differences that contribute to interpersonal relationships, adding, “to accept the risks associated with the type and depth of the interdependence inherent in a given relationship.” This addition is particularly relevant when considering consumer–health professional interactions, due to the inherent power differential in the relationship. Institutional trust is defined as “the expected utility of institutions performing satisfactorily” (Mishler & Rose, 2001) and is influenced by personal experiences within the institution or its wider system, as well as social and cultural norms and public perception. Reflecting on the medical profession's volatile history with BPD and those given this diagnosis, in terms of stigma, dismissal and mistreatment, as well as directly or indirectly experienced stigma and misunderstanding from the wider community, it is understandable that consumers may be hesitant to trust health professionals or institutions, particularly psychiatrists and mental health nurses (MHNs) in an inpatient environment. With the current ease of sharing information via the Internet through platforms like social networking and forums, consumers are also able to connect more easily with one another, and share stories of lived experiences, which may also affect their views on the trustworthiness of healthcare systems and workers. Considering emotional instability and experiencing emotions intensely are characteristic and diagnostic features of BPD, it can be suggested that each interaction holds even more importance for that individual's overall trust in health care than people with other diagnoses.

The benefits of effective treatment for people diagnosed with BPD extend beyond the individual and their social network to reducing the economic burden associated with ineffective BPD management. Personality disorders in general account for a high economic cost to society, and people with a BPD diagnosis are significantly more likely to use all types of psychiatric services and see a greater number of specialists (Bender et al., 2001; Jackson & Burgess, 2004; Meuldijk et al., 2017; Soeteman et al., 2008). An Australian study by Lewis et al. (2018) on acute presentations to psychiatric services found that patients with personality disorders were 2.3 times more likely to re‐present within 28 days of their first presentation than others and that personality disorders were second only to psychoses in increasing the rate of admission factor. This study also found that over a fifth of patients presenting to emergency, and a quarter of inpatients, had a personality disorder diagnosis. Equipping people diagnosed with BPD with the skills and support to successfully manage their emotions and engage in society through effective, evidence‐based treatments leads to better quality of life for consumers, resulting in decreased need for emergency and hospital services, and offering a potential solution to the currently high economic cost of BPD management.

The National Health and Medical Research Council (NHMRC) released Guidelines for the management of people with a diagnosis of BPD in February 2013, with evidence‐based recommendations relating to diagnosis, treatment options, ongoing management, utilization of healthcare services, support of carers and family, as well as general principles for care. In addition to the above sections, they contain management and crisis management plan templates. These Guidelines are of specific relevance to mental health professionals involved in regular care of people diagnosed with BPD, such as MHNs, in guiding their practice and decision‐making. They also offer advice more broadly to other health professionals such as general practitioners (GPs) and allied health who may have responsibility for providing care to people diagnosed with BPD, with a condensed version of the Guidelines available for easy reference. Coincidentally, a survey investigating experiences of care by people with a BPD diagnosis was administered in 2011, before these Guidelines were released, and again in 2017 (Lawn et al., 2017; McMahon & Lawn, 2011). The term consumer is primarily used throughout this paper, to describe individuals diagnosed with BPD who seek care from health services.

### 1.1 Rationale

Although this survey was not administered with the purpose of investigating the Guidelines in any way, and therefore no direct causal relationship can be drawn, the two time points at which it was delivered does provide an opportunity to assess changes in consumer experiences over this time. As such, this research can provide a useful insight into whether, given the release of the NHMRC Guidelines in this time, treatment has improved from the consumer perspective. It also provides an exemplar to guideline developers, researchers, clinician services and clinicians, and consumer advocacy groups in other countries that may offer insights and therefore help support improvements in BPD care to practice, more broadly. As one of the primary aims of the Guidelines is to improve the delivery of BPD management, and therefore the consumer perspective on management, the authors believe it is reasonable to examine the changes in consumer opinion over this time as one way to assess the potential impact of the Guidelines in practice. The impact of the Guidelines on Australian consumers has not been addressed in the existing literature. The authors have analysed data from the Australian context, as their primary area of residence and practice, and acknowledge the potential for differences in other geographic locations. A study by Simonsen et al. (2019) comparing BPD guidelines across several European countries noted important contradictions between recommendations in relation to diagnosis, length and setting of treatment, and use of pharmacological treatments, but consensus on recommending the use of psychotherapy. Of note, these authors strongly recommended the inclusion of consumer perspectives in guideline development and review. The qualitative data from the Australian consumer surveys have not previously been analysed and as such is an important source of information on consumer perspectives on how they Australian guidelines are faring that deserves thematic analysis and discussion.

### 1.2 Aims/Objectives

The aims of this paper were to understand consumer perspectives regarding BPD management, and how these have changed between 2011 and 2017, as well as commenting on how the NHMRC BPD Guidelines are faring in practice, given their release in 2013.

## 2 METHODS

### 2.1 Design

Data presented in this paper are drawn from two online surveys, one to people with a diagnosis of BPD and the other to their carers (predominantly family providing unpaid informal support), administered on two separate occasions across Australia, in 2011 and 2017. The 2011 survey was developed and delivered by the Private Mental Health Consumer Carer Network (PMHCCN—now known as Lived Experience Australia) as part of its Chair, Janne McMahon's, works with the Australian Commonwealth Government's BPD Ministerial Expert Reference Group (BPDERG). The PMHCCN delivered the surveys again in 2017 as part of its national mental health advocacy work. The surveys were developed to gather information from people with a diagnosis of BPD, and their careers, on their perceptions of care and experiences with seeking support in public and private health services. The results of the carer survey are not discussed in this paper.

### 2.2 Recruitment and data collection

The consumer survey was delivered online via SurveyMonkey across all Australian states and territories in May–June 2011, and June–July 2017, through 29 consumer and carer mental health networks via their electronic and paper communications, including 20 clinical mental health and non‐government community organizations. The survey contained 75 questions, covering demographic details, diagnosis and treatment, impacts, suicide/self‐harm, and contact with GPs, mental health services, hospitals and other supports. Most questions sought Likert‐rated responses, and for several questions, participants were also offered an opportunity to provide further comments, meaning qualitative and quantitative data were collected. Quantitative results of the survey have been published elsewhere (Lawn et al., 2017; McMahon & Lawn, 2011); therefore, this paper will focus on comparing and reporting qualitative results from the surveys, with particular regard to what they say in relation to the key practice domains noted as important in the NHMRC Guidelines.

Participants in the 2011 survey included 153 Australians with a diagnosis of BPD, with 60.1% of respondents answering all questions and remaining participants being selective in their responses. In 2017, there were 424 participants in the survey, and 16.27% of respondents answered all questions, with other participants being selective in their responses. Although the percentage of respondents answering all questions dropped significantly from the first to second survey, where applicable, 2017 survey questions were consistently answered by approximately three‐quarters of the total participants; that is, there were approximately 300 responses to each survey question. Likewise, in the 2011 survey, there were approximately 100 respondents to each question across the survey, where applicable.

### 2.3 Ethics

Permission to access the data and conduct this study was granted by the PMHCCN. Participation in the surveys was open to anyone who identified as having a BPD diagnosis, and informed consent was assumed via participation in the voluntary online survey. Ethical considerations for the original 2011 survey were informed by consultation with the PMHCCN National Committee of consumers and carers, and BPD academics linked with the BPDERG. Ethics approval for the current study was granted by the Social and Behavioural Research Ethics Committee, Flinders University (No. 7613).

### 2.4 Bias and reflexivity

The authors acknowledge the potential for bias in any interpretation of meaning from qualitative data, particularly given the mental health consumer systematic advocacy roles played by some of the authors. We addressed this through the robust team discussion during the analysis processes described below and by presenting our preliminary findings for scrutiny to mental health peers at the Royal Australian and New Zealand College of Psychiatrists Annual Congress (2019). The audience at this presentation also included some individuals involved in the development of the Guidelines, who were given opportunity to comment and discuss their thoughts with some of the authors.

### 2.5 Data analysis

Survey responses were analysed using summative content analysis, which involves subjective interpretation of the data through systematic classification and coding to identify themes or patterns (Hsieh & Shannon, 2005). The first author undertook data analysis by reading and re‐reading respondents' qualitative responses, word by word. They then undertook formal analytic memo writing to begin formulating general impressions about participants' responses, checking back and forth across the data, highlighting phrases with similarities and differences in perspectives as part of preliminary organization of the data into themes. Two members of the research team met regularly to discuss and debate the tentative and final groupings, checking them against the original data sets, also to ensure accuracy of context was captured. Specific examples of responses that exemplified each theme were discussed and agreed upon. The themes which emerged related broadly to the sections in the shortened version of the Guidelines—“Caring for People with Borderline Personality Disorder: A Reference Guide for Health Professionals” (NHMRC, 2013; NHMRC, 2013), and as such, the research team also agreed to present results under these subheadings.

## 3 RESULTS

De‐identified direct qualitative quotes from survey respondents have been selected which best demonstrate the themes and reflect the dominant responses across the data. The terms “respondent,” “consumer” and “patient” are used interchangeably, where appropriate, to denote people with a diagnosis of BPD who responded to the surveys, and when talking about the experiences of people with a BPD diagnosis generally.

### 3.1 Transparency and communication regarding BPD diagnosis

The Guidelines recommend that people diagnosed with BPD should be told their diagnosis, have the diagnosis and symptoms explained to them and related to their own experiences, with emphasis on the fact it is not their fault, and that effective treatments are available. The data highlighted two main problems consumers faced when being diagnosed with BPD: the health professional making the diagnosis withholding this information from the consumer and poor explanations of BPD at the time of diagnosis (Table 1).

**Table 1:** Transparency and communication regarding BPD diagnosis

2011	1. “…I got hospital files and found out they said I had BPD when I was 18 I had no idea!! I feel really angry about this. People MUST explain diagnosis not just hand them out. I didn't find out what BPD was until I was 38 years old and a therapist explained it…”
	2. “I had a hospital stay 16 years ago and was diagnosed with BPD – this has never been explained to me and I have been treated for depression ever since.”
2017	3. “If I had been properly assessed when I was first diagnosed with a mental health condition, I wouldn't be in this situation now, people might take me seriously, and I could have started ACT and DBT much sooner. I was 17 at the time, and no psychiatrist would have diagnosed me with BPD.”
	4. “I was diagnosed with BPD at age 19 but had already brought up the possibilities to several psychologists and the school counsellor I had during my 15–18 years of age. I was diagnosed with depression at age 14 but felt there was much more so I did my own research to gain some understanding. I brought up my concerns with these professionals but they didn't consider the possibilities (due to stigma, lack of knowledge or that I was good at masking my problems) and as a result I received inadequate treatment. It was only until I was admitted to hospital for BPD‐related issues at 18 and was referred to another psychologist that the possibility of me having BPD was taken seriously.”
	5. “Psychiatrist diagnosed BPD but didn't tell me – only found out when she made a report to AHPRA re suicide attempt/alcohol use, still see her but will never trust her again (didn't tell me about the report either, that was left to my psychologist).”
	6. “Given a diagnosis of BPD in 1975 by a psychiatrist, I was 12. Since then I have not received any information and didn't know what was the point of the label.”

Respondents in 2017, in general, appeared to be given their BPD diagnosis earlier than was the experience of 2011 respondents; however, comments regarding the negative impact of not receiving their diagnosis early enough prevailed across both samples. In 2017, there was a sense that respondents were more aware of their own behaviour as likely to be a mental health problem. They also seemed more health literate about the characteristics of BPD, leading them to suggest the diagnosis to health professionals, but unfortunately, they often felt that these thoughts were not considered or taken seriously, leading to delay in diagnosis and treatment. Comments about younger respondents not being taken seriously due to their age were also prevalent in both data sets, despite the Guidelines stating that although the diagnosis is not recommended for prepubescent children, assessment for BPD should be considered in people presenting with the relevant signs from age twelve.

Only 50.69% of respondents in 2017 felt that the health professional making their diagnosis had explained it to them at the time in a way they understood. Respondents in 2011 and 2017 commented repeatedly on the frustration they felt at inadequate explanation of their diagnosis. Respondents stressed that this poor explanation meant that they were not equipped to access appropriate treatment, leading to poorer quality of life and increased use of emergency services during crises.

Psychiatrists most frequently made the BPD diagnosis, as reported in both 2017 (72.68%, *n* = 258) and 2011 (76.9%, *n* = 90) (Lawn et al., 2017; McMahon & Lawn, 2011). The comments below suggest that some psychiatrists, when communicating a new BPD diagnosis to a patient, have not adequately explained the diagnosis, contextualized it for the patient in terms of their experiences, or offered appropriate treatment options. The data suggest that this issue is improving, as comments referring to withheld or poorly explained diagnoses largely referred to past situations. Particularly examining the 2017 data set, comments describing problems with new BPD diagnoses being withheld were almost non‐existent, and reports of poor explanations of the diagnosis decreased compared with 2011 data.

### 3.2 Interpersonal approach demonstrated by health professionals in BPD management

The Guidelines state that health professionals working with people diagnosed with BPD should listen to the person's experiences and take their feelings seriously, whilst being respectful, caring, compassionate, consistent, reliable and non‐judgemental. Comment 8 (Table 2) highlights the negative effects on consumers when they perceive they are not being treated respectfully. The psychiatrist explicitly addressing the power imbalance in the therapeutic relationship resulted in increased trust and understanding in comment 7, and comments 9 and 11 show the positive impact of the psychologist listening and making efforts to understand the respondent's experience. However, it was still apparent in 2017 that many consumers felt dismissed, misunderstood and in some cases demonized, by their health professionals, and many still expressed that they felt they were not taken seriously. The idea of BPD not being a “real” mental illness was present, and continued to be so in 2017, despite the Guidelines specifically addressing the need to understand BPD as a legitimate use of health services. This was especially reported by respondents regarding hospital admission and in crisis situations, scenarios in which MHNs play an integral role in face‐to‐face service delivery.

**Table 2:** Interpersonal approach demonstrated by health professionals in BPD management

2011	7. “I want to help others and in return others help me. This limits the damage of very uneven power, which destroys many so‐called 'therapeutic' relationships. My present psychiatrist checks the power stuff with me constantly. He asks things like, ‘was that patronising?’ and sometimes I say, ‘yes’.”
	8. “Derogatory comments from paramedics and other health staff, psych nurses and psychiatrists telling me I don't have a mental illness, or not a 'real' mental illness, health professional ignoring physical health symptoms because of my mental health diagnosis.”
2017	9. [Re: contributed most to recovery] “A very supportive partner and to a degree, a psychologist who is willing to listen, learn and understand.”
	10. “I see my psychiatrist as my first port of call for mental health problems, not my GP. I get more time per session (45 min) so we can talk about more things than a 10‐min GP session.”
	11. [Re: health professional most effective at helping to understand feelings] “What about professional counsellors? …sit with people in distress rather than judge, label, direct. my therapist is psychologist but uses very little of her psych training. she draws on attachment theory and her authentic, compassionate connection. therapeutic relationship heals…”

### 3.3 Managing crises, self‐harm and suicidal behaviour

The Guidelines state that all health professionals should recognize that BPD treatment is a legitimate use of healthcare services and that having a BPD diagnosis should never be used as a reason to refuse health care to an individual. However, they also recommend that hospital admission should not be used as standard treatment for BPD. Brief admission to acute psychiatric inpatient facilities should only be considered if the person is at significant immediate risk of harm or has a co‐occurring mental illness. It seems that distinguishing the level of immediate risk to the consumer in emergency presentations causes significant problems for health professionals and consumers (Table 3). Many comments from 2011 and 2017 describe respondents feeling as if they were at imminent risk of self‐harm and unable to manage on their own, but assessment from health professionals, particularly paramedics, MHNs and psychiatrists, did not align with the respondent's own beliefs at the time.

**Table 3:** Managing crises, self‐harm and suicidal behaviour

2011	12. “The private psychiatrist felt that hospitalisation was not needed. She was however not in my skin.”
	13. [Re: private hospital admission refusal] “When I got suicidal again after I left the inexperienced shrink [psychiatrist] I asked the new one if I ‘should’ go. He said he knew I could get through it. I did!”
2017	14. “Psychiatrist didn't think it important to be hospitalised but I was suicidal and have trouble getting my feelings across.”
	15. [Re: private hospital admission refusal] “At first they were going to admit me as I was withdrawing from medication and having severe reaction. The ER doctor went off and spoke to head of psychiatrics and I was refused on the grounds that I have BPD.”
	16. “Help is always offered by my psychiatrist and psychologist. My problem is l don't like to ask for help, especially when in crisis because of the fear of being put back into hospital where l and my family have to fight to for me to stay there for more than 2 days even though l am acutely suicidal.”

Comment 13 points to the importance of stable management, when this person transitioned to a new psychiatrist and experienced suicidal thoughts, the support of the new psychiatrist meant they avoided hospitalization and confidence in their ability to control their own emotions increased. A recommendation of the Guidelines includes explaining to the respondent that it is not feasible to depend on the mental health service or GP to be available at all times, and to help them use a problem‐solving approach to identify practical alternatives in a crisis. From these data, it is evident that there continues to be significant use of hospital emergency departments for individuals diagnosed with BPD in a crisis, rarely resulting in positive therapeutic outcomes. MHNs are well placed to provide consistent and routine care to enhance stability in the consumers' BPD management. Nurses are also able to undertake training in a wide range of areas, including in providing psychoeducation to patients, helping to build consumers' capacity and autonomy regarding day‐to‐day management of distress and BPD symptoms.

### 3.4 Managing trauma

The potential link between trauma and BPD is addressed in the Guidelines, and the high prevalence of comorbidities (such as PTSD) linked to previously reported trauma in both data sets highlights this (Lawn et al., 2017). The Guidelines specify that clinicians should avoid re‐traumatizing patients and that questions about past adverse experiences should be handled sensitively. Comments 17–18 (see Table 4) highlight the distress caused by a health professional exposing trauma without patient guidance and sensitivity.

**Table 4:** Managing trauma

2011	17. “The psychiatrist I had for two years did not help at all she tried to counsel me and I was going weekly as well as my psychologist and this made me worse before I realised she didn't know what she was doing. It does NOT help talking about abuse. I have the new shrink [psychiatrist] who knows BPD and my psychologist who is the expert here on BPD and I am doing much, much better.”
2017	18. “Psychiatrist did help with medication and has done her best in other ways – but if anything is more likely to trigger distress than to help resolve it as she goes in too hard. Psychologist is much better at dealing with childhood trauma and has been willing to learn how best to assist me.”

### 3.5 Management plans

The Guidelines specify that every individual with BPD should have a tailored management plan, developed in collaboration with them, and that their family/carer/partner should be involved if the individual consents and this is in their best interest. This should also include a brief and clear crisis plan, and these plans should be shared with all health professionals involved in the person's care. Access to a management plan could be of huge assistance to health professionals working with people diagnosed with BPD, especially in crisis or with health professionals new to the patient. However, comments 19–20 (see Table 5) demonstrate that these respondents perceived their management plans as more harmful than helpful, and they were not invited to collaborate on how they wanted to be treated. The damage caused by plans made without their consultation was explicitly stated in these comments, including one respondent stating that their plan increased their desire to self‐harm (comment 20). Interestingly, there were very few comments explicitly mentioning care plans in either data set, and no mention of any BPD‐specific plans.

**Table 5:** Management plans

2011	19. “I think that if my hospital plan was removed then I would be more happy to attend a hospital or get the help I need when it's needed. At the moment, they put a plan in place that if I turn up before acting then I will have access to a mental health nurse. If I turn up after then they will treat me medically but will not be allowed to get the emotional help, which means I leave in the same headspace and do the same thing over again. Now I just don't even attend even if I know I have done something stupid. It's frustrating as I continually tell my psychiatrist this but it doesn't change.”
	20. “Now days they sometimes have these ridiculous contracts and you are sometimes allowed in hospital for three days when you choose but you get kicked out if you self‐harm. They reckon these contracts and Treatment Plans are written collaboratively. I can't be bothered arguing any more. They are going to write what they want to write anyway so what's the point. The contracts are demeaning and they just make me want to self‐harm.”
2017	21. [Re: contributed most to support recovery] “Psychiatrist, DBT, Advanced DBT, GP Chronic Illness Management Plan.”

### 3.6 Barriers to treatment

There were many comments from both 2011 and 2017 regarding barriers to treatment, most of which related to a lack of available specialized services. Although there was a sense that appropriate services were more plentiful in 2017, they were still either too far away, too expensive or the wait list was too long for respondents to utilize those services (Table 6). Another barrier, highlighted by comment 26, is the exhaustion that searching for a new therapist can cause consumers, when their regular health professional takes leave or retires. Adequate training and support for MHNs, psychologists, and other allied health, may help to alleviate this particular issue, by altering the general perception that BPD treatment is delivered primarily by psychiatrists, when in fact, training in multiple effective BPD treatment options is available to other health professionals (e.g., dialectical behaviour therapy (DBT), cognitive behaviour therapy (CBT) schema‐focused psychotherapy (SFP)). Whilst barriers to treatment persisted as a dominant theme across both data sets, the 2017 survey data indicated that individuals were seeking out more of these evidence‐based psychological treatments. Comparison of the quantitative data clearly showed a shift towards psychologists as primary treatment providers, with 76.2% (*N* = 80) of respondents primarily seeking support from a psychiatrist in 2011, to 2017 where psychologists were the primary support professional for 84.19% (*N* = 245) of respondents (Lawn et al., 2017; McMahon & Lawn, 2011). Respondents to the 2017 survey also appeared to have great awareness and use of DBT group therapy, though there were many comments about long waiting times for access to these services.

**Table 6:** Barriers to treatment

Physical barriers	2011	22. “Services are available but getting myself into them is a major problem.”
		23. “My psychiatrist has taught me how to manipulate the mental health system to my better advantage. It is a really useful skill. For example, if I am admitted to a public psychiatric unit he generally emphasises that I have Bi‐polar and underplays the Complex Post Traumatic Stress Disorder. He tells me which psych nurses are the good eggs and tries to manipulate so they become my contact nurses if at all possible. This brings peace I allow myself to rest and not be concentrating so hard on staking a claim by self‐harming and being desperate. It is very easy to be seen to be bad rather than mad when you have a diagnosis like BPD.”
	2017	24. “Psychiatrists are extremely difficult to access in a regional area.”
		25. “More needs to be done to provide trained and experienced psychologists and psychiatrists in regional and rural areas and to improve the training of mental health nurses in understanding the immediate care needed by sufferers of BPD when in crisis.”
Emotional barriers	2011	26. “I found my psychiatrist myself, long tormented process of eliminating ones who I don't feel comfortable with.”
	2017	27. “Psychiatrist referrals (multiple in the last two weeks) have been unsuccessful as all are full and very, very few specialise in the area. There is a severe drought for BPD sufferers.”
Financial barriers	2011	28. “I say if I had to rely on the public hospital and without my private psychiatrist I say I would be dead.”
		29. “I have been fortunate enough to have the money to access these services. Not so for everyone. I don't know how I would have recovered if I was on a pension.”
		30. “Long term access to a psychiatrist to see and talk about [effective BPD] treatments available – it's too expensive for me to afford it.”
	2017	31. “Also having more access to psychiatrist, affording medication and allied health providers has given me a better quality of life and less need for hospitalization.”
		32. “Only [can afford treatment] because my psychiatrist only charges me $20 over the Medicare rebate so I can continue to get some assistance.”

## 4 DISCUSSION

Effective, evidence‐based treatments are available which assist people with a diagnosis of BPD to recovery, so they can succeed in personal relationships, careers and other aspects of life. However, this Australian study showed, despite the presence of national Guidelines, that individuals with a diagnosis of BPD continue to experience frustration and stress when navigating healthcare systems to seek treatment and help understanding their experiences and feelings. Reflecting on the qualitative data from these surveys, which report a diverse yet inconsistent range of helpfulness from different clinicians, there is scope to improve education and practice across each and all aspects of the Guidelines. Stigma from health professionals and the general public, frequent emergency presentations and poor access to services can make it difficult for people with a BPD diagnosis to manage their emotions and pursue their goals. Following the Guidelines' principles for treating people diagnosed with BPD is an important first step to eliminating stigma within health services, and creating positive interactions that contribute to individuals' overall treatment. MHNs are uniquely positioned to champion this change in their regular day‐to‐day interactions with patients, but must be properly supported by management, with adequate training and clinical debriefing support, to do this effectively. The Guidelines are also in need of review to ensure they are in‐line with the latest evidence, as they have not been updated since their creation in 2012. The findings of this study offer an opportunity for those responsible for developing and implementing similar guidelines in other countries to recognize the need to support their successful implementation into practice.

Consumers across both data sets frequently reported distress when seeking help in crisis, such as self‐harming, suicidal thoughts or attempts. Inappropriately slow responses to physical injuries and psychological distress, lack of empathy and poor listening skills seemed to be amplified in the emergency department and acute inpatient setting. Respondents most often referred to paramedics, MHNs and emergency department staff as particularly unhelpful, more so than psychiatrists; however, respondents are aware of the control that psychiatrists have over the decision of whether they will be admitted or not (Ring & Lawn, 2019). Considering the concepts of interpersonal and institutional trust discussed earlier, the negative effects of such an experience on the individual's recovery, and implications for future interactions with healthcare services can be appreciated. It is clear from the data that many respondents, when seeking help by presenting to an acute health service in crisis, felt that they did not receive the help they required, or were actively ignored and/or mistreated by health staff at the time. A key recommendation of the Guidelines is that all individuals diagnosed with BPD have a person‐centred management plan, developed collaboratively between consumer, carer/family and health professionals. BPD‐specific management plan and crisis management plan templates are provided in both versions of the Guidelines, and the finalized plans are designed to assist health professionals who are unfamiliar with the consumer to access information about the consumer's needs and preferences, so they can provide appropriate treatment and support to the individual and their family/carers. Respondents rarely referred to management plans in either data set, which may reflect the survey itself, as it was not designed to gain data specifically relating to management plans. However, where references to plans were made, it was in an overwhelmingly negative way, with multiple respondents highlighting that their plans were not developed with them collaboratively and were instead forced upon them as control measures. The importance of collaboratively developed management plans is noted in the Guidelines, and specialist BPD services like Spectrum, Victoria, Australia, use methods such as co‐authoring to ensure consumers feel involved and in control of their BPD and its management (Mental Health Professionals' Network, 2018). These data show that consumers did not feel involved in the development of their management plans and did not see them implemented in a way that contributed to their recovery. Respondents described limitations to treatment access dependent on their actions and expressed fear of detainment if they presented to an emergency department with self‐harm injuries. The exact nature of these plans cannot be determined from these data, and it is possible that some of the plans mentioned refer to Involuntary Treatment Orders (ITOs), informal “contracts” with their regular doctor, or other circumstances, which serve a different purpose than the plan templates in the Guidelines.

What is clear from the data is that management plans, for many consumers, are not present in the way in which the Guidelines recommend—in that a collaboratively written guiding document is available to the consumer, their family/carers, and all health professionals and services they interact with, which outlines how to address their needs and manage distress or crisis. Accessible, well‐made management plans, outlining agreed responses and actions for when the person with BPD seeks help for their distress and self‐harm, would be of particular use to health professionals providing care in acute or crisis settings, such as MHNs, who comprise a large proportion of this workforce. For health professionals more generally, well‐written and regularly updated management plans would help alleviate concerns regarding a lack of understanding of BPD management in general. More research into the development and use of management plans from both consumer and health professional perspectives would add greatly to this conversation, providing a potential solution to poor communication and management in crisis.

The findings clearly show that consumers want to receive comprehensive, holistic and evidence‐based support, including psychotherapy (Carrotte et al., 2019). The data also highlight a shift in consumer preference from psychiatrists in 2011, to psychologists in 2017, as their main treating health professional. It is impossible to define the exact reason for this from the data, but one possible explanation is a view amongst consumers that psychiatrists' role is to diagnose and prescribe medications, whereas the role of psychologists is to assist with understanding emotions and behaviours, as suggested by one of the respondents. In Australia, GPs can also prepare a Mental Health Care Plan for eligible patients, allowing the consumer access to up to 20 Medicare‐subsidized sessions with a psychologist per year under the Better Access Scheme (Services Australia, 2020), which may explain increasing use of psychologists. An increasing awareness of and interest in BPD generally may also extend to psychology, mental health nursing and other disciplines arguably more able to provide holistic care, and as services are highly in demand, more psychologists are training to provide treatments for BPD such as DBT, CBT, SFT and others. Albeit this has not solved the continuing difficulty consumers face trying to access services, as long waiting lists for individual and group therapy programmes, unaffordable therapies and long commutes to city‐based services continued to be noted as barriers to treatment in both data sets. MHNs can also undertake further training to provide these therapies, improving access for consumers. Particularly in rural areas where specialized mental health services are often less robust and a psychiatrist may not always be available, MHNs would be well positioned to coordinate and lead group and individual therapy programmes, improving continuity and access to regular treatment.

It is time for a review and update of the Australian Guidelines, and a national strategy and funding for their implementation throughout Australia's healthcare systems, such that health professionals at all levels of patient interaction are trained to understand and respond to the needs of people diagnosed with BPD, starting with those at ground level—particularly MHNs, paramedics and emergency department staff. Achieving this would require a commitment from higher‐level policymakers and government, to ensure that people with a BPD diagnosis are treated effectively and appropriately when interacting with healthcare services. This commitment should be one they are eager to make, to reduce the increased economic costs and inpatient admission time associated with ineffective BPD treatment (Bender et al., 2001; Jackson & Burgess, 2004; Lewis et al., 2018; Meuldijk et al., 2017; Soeteman et al., 2008).

Training for health professionals outside of specialized BPD services is lacking, and what exists is highly variable in its success. More research is needed into education programmes and ways to implement training on appropriate conduct and skills for health professionals when working with people diagnosed with BPD. Ongoing work by advocacy groups and government campaigns is needed to eliminate stigma towards people with a BPD diagnosis, and fight for adequate funding and services. Recent successes in South Australia demonstrate what can be achieved with this attitude and commitment, with the launch of the Borderline Personality Disorder Collaborative (BPD Co) on 7 June 2019. This centre provides coordinated care, tailored to the needs of individual consumers, carers and clinicians, as well as collaborating with universities and others in a research role, to further inform evidence‐based practice and continue to improve service delivery. BPD Co also has a training and education programme, aiming to upskill health professionals and staff involved in service delivery, carers and families, and eventually the wider general community (SA Health, 2019). BPD is a complex and often debilitating health condition for individuals and their families, but with awareness, education and accessible, evidence‐based, effective treatments, better outcomes for individuals with this manageable condition can be achieved.

## 5 LIMITATIONS

It is not possible to determine how many consumers the invitation was forwarded to, therefore making it impossible to determine an overall response rate. Additionally, survey respondents were not a random sample of the population of people with a diagnosis of BPD, as they self‐selected by virtue of choosing to respond to the survey; therefore, the extent to which the conclusions drawn from these surveys represent the wider population of Australians with a BPD diagnosis depends on whether response bias exists and its influence. The surveys did not specifically ask about knowledge or use of NHMRC BPD Management Guidelines, and therefore, no direct causal relationship can be attributed to the Guidelines for changes observed. There was no way to identify and match responses for respondents who completed the survey in 2011 and 2017, as they were completely anonymous. This was done to encourage participation and minimize potential stigma. It was also not ascertained if 2017 respondents had received their diagnosis since 2011, or were responding to experiences prior to 2011. However, original researchers did their best to convey and ensure survey questions were asked with the intention that participants would recall their “current” experience. Inconsistent and variable response rate to survey questions was a further limitation. However, whilst only approximately 16% of respondents answered all questions in the 2017 survey, where applicable, there were approximately 300 respondents to each survey question. Likewise, whilst approximately 60% of 2011 survey respondents completed all questions, there were approximately 100 respondents to each question across the survey, where applicable.

## 6 RELEVANCE TO MENTAL HEALTH NURSING STATEMENT

Caring for people with a BPD diagnosis is an important part of mental health nursing. This paper analyses consumer‐reported qualitative survey data from 2011 and 2017. These comparative data sets offered an opportunity to examine the impact of the National Health and Medical Research Council BPD Management Guidelines, released in February 2013, on practice. Although a direct causal relationship between the Guidelines and changes observed in the data is not clear, these time points provided an opportunity to consider the Guidelines influence, for improved practice by mental health nurses and other health professionals, and outcomes for people diagnosed with BPD.

## CONFLICT OF INTEREST

The authors declare that there is no conflict of interest.

## AUTHOR CONTRIBUTIONS

Miss Proctor and Professor Lawn formulated the study design; Miss Proctor then undertook the analysis, with input from Professor Lawn and critical comment from Ms McMahon.

## ETHICAL APPROVAL

Approval granted by the Social and Behavioural Research Ethics Committee, Flinders University (no. 7613).

## Data Availability

The data that support the findings of this study are available from Lived Experience Australia. Restrictions apply to the availability of these data, which were used under licence for this study. Data are available from the authors with the permission of Lived Experience Australia.
